# Metformin targets multiple signaling pathways in cancer

**DOI:** 10.1186/s40880-017-0184-9

**Published:** 2017-01-26

**Authors:** Yong Lei, Yanhua Yi, Yang Liu, Xia Liu, Evan T. Keller, Chao-Nan Qian, Jian Zhang, Yi Lu

**Affiliations:** 1 Key Laboratory of Longevity and Ageing-related Diseases, Ministry of Education, Nanning, 530021 Guangxi P. R. China; 2 Center for Translational Medicine, Guangxi Medical University, 14th Floor, Pharmacology and Biomedical Sciences Building, No. 22 Shuangyong Road, Nanning, 530021 Guangxi P. R. China; 3 School for International Education, Guangxi Medical University, Nanning, 530021 Guangxi P. R. China; 4 Department of Urology and Pathology, School of Medicine, University of Michigan, Ann Arbor, MI 48109 USA; 5 Department of Nasopharyngeal Carcinoma, State Key Laboratory of Oncology in South China, Collaborative Innovation Center for Cancer Medicine, Sun Yat-sen University Cancer Center, Guangzhou, 510060 Guangdong P. R. China

**Keywords:** Metformin, Signaling pathway, Cancer stem cell, Cancer

## Abstract

Metformin, an inexpensive and well-tolerated oral agent commonly used in the first-line treatment of type 2 diabetes, has become the focus of intense research as a candidate anticancer agent. Here, we discuss the potential of metformin in cancer therapeutics, particularly its functions in multiple signaling pathways, including AMP-activated protein kinase, mammalian target of rapamycin, insulin-like growth factor, c-Jun N-terminal kinase/mitogen-activated protein kinase (p38 MAPK), human epidermal growth factor receptor-2, and nuclear factor kappaB pathways. In addition, cutting-edge targeting of cancer stem cells by metformin is summarized.

## Background

**Fig. 1:**
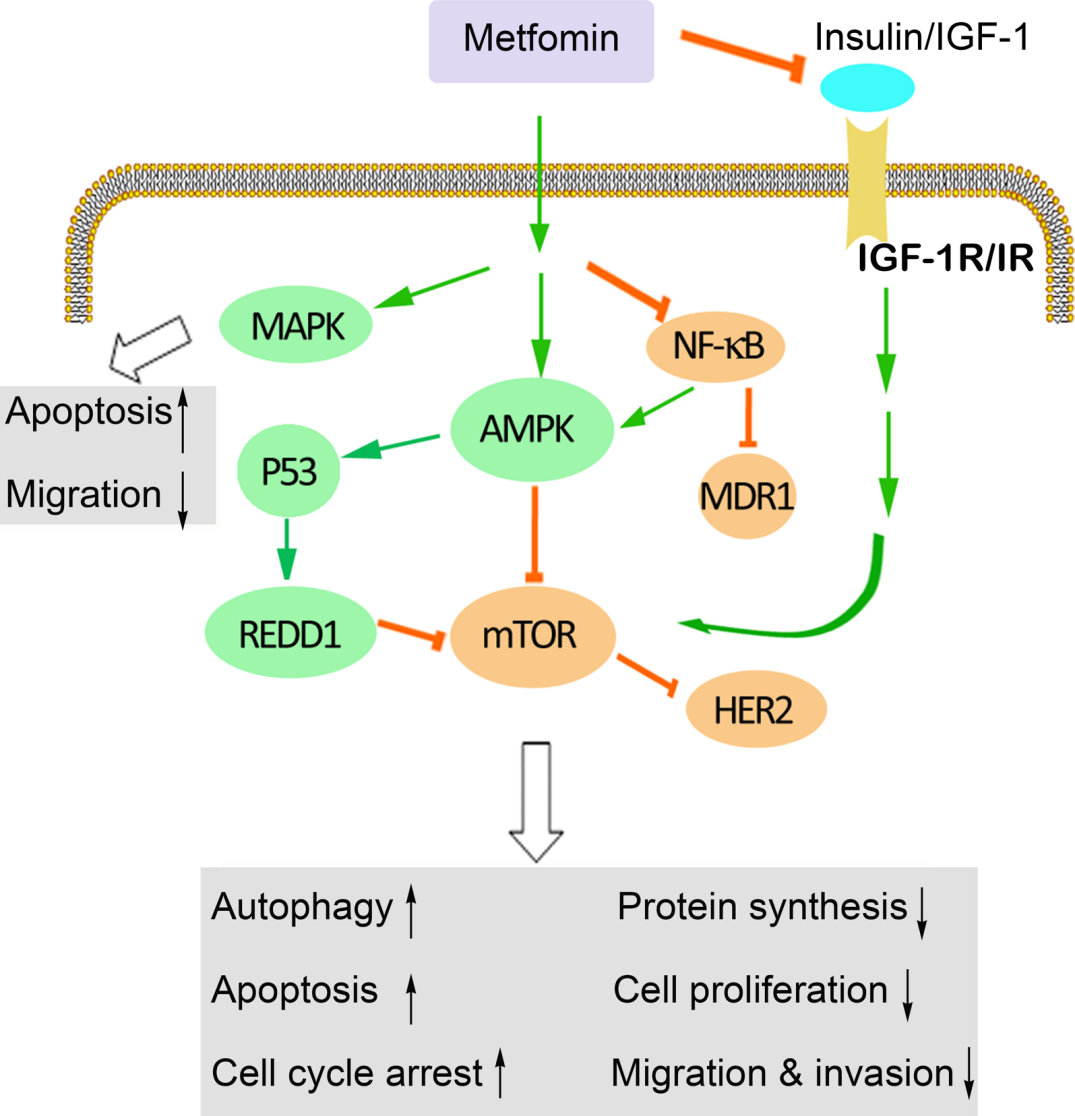
Signaling pathways through which metformin acts in cancer. *IGF*-*1* insulin-like growth factor-1, *MAPK* mitogen-activated protein kinase, *REDD1* regulated in development and DNA damage 1, *AMPK* adenosine monophosphate-activated protein kinase, *mTOR* mammalian target of rapamycin, *NF*-*κB* nuclear factor kappaB, *MDR1* multidrug resistance 1, *HER2* human epidermal growth factor receptor-2, *IGF*-*1R* IGF-1 receptor, *IR* insulin receptor

Metformin (1,1-dimethylbiguanide hydrochloride), a USA Food and Drug Administration (FDA)-approved biguanide derivative and the most widely prescribed antihyperglycemic drug, is used as first-line therapy for diabetes mellitus type 2. Metformin reduces blood glucose levels by inhibiting hepatic glucose production, increasing glucose uptake and utilization by the skeletal muscle, reducing insulin resistance in peripheral tissue, and suppressing gluconeogenesis in the liver [1–4]. Interestingly, metformin attracted increasing interests in recent years due to its anticancer effects [5–10]. The drug has been demonstrated to reduce the development of prostate cancer [11], lung cancer [12], breast cancer [13], esophageal cancer [14], colon cancer [15], and melanoma [16]. Several preclinical studies have reported that metformin reduced cell proliferation, induced apoptosis, and caused cell cycle arrest in vitro and also reduced occurrence and growth of experimental tumors in vivo [17–19]. Metformin can also be used as a sensitizer or be combined with conventional chemotherapeutic agents and radiotherapy to combat cancer [20–24]. Moreover, metformin plays an important role in targeting cancer stem cells (CSCs) [25] and reversing the epithelial-mesenchymal transition (EMT), a critical process in cancer metastasis [26]. The possible signaling pathways involved in the anticancer effects of metformin are outlined below and demonstrated in Fig. 1 and Table 1.

**Table 1:** Metformin targets multiple signaling pathways in cancer

Proposed mechanism	Functions	Tumor type/model	References
AMPK-dependent	Inhibition of cell mitosis and proliferation	Human carcinoma tissues and human cancer cell lines	[29]
	Up-regulation of the p53–p21 axis and down-regulation of cyclin D1	T-cell acute lymphoblastic leukemia	[30–32]
	DNA synthesis	Pancreatic cancer	[34]
	Growth inhibition and G_0_/G_1_ cell cycle arrest	Lymphoma cells	[36]
	Cell apoptosis	Acute lymphoblastic leukemia	[36]
	Suppression of multidrug resistance 1 gene activation	Breast cancer	[37]
AMPK-independent	REDD1, a negative regulator of mTOR, mediates cell cycle arrest and cyclin D1 decrease	Prostate cancer cells	[39]
	Induced apoptosis	Human ovarian cancer cells	[40]
Suppression of mTOR	Inhibition of global protein synthesis and cell proliferation	Breast cancer	[54–56]
	Repression of oncogenic mRNA translation	Leukemia	[30, 32]
		Lung cancer	[59, 60]
	Inhibition of cell growth and induction of apoptosis	Breast cancer	[61, 62]
	Prevents the development of carcinogen-induced premalignant lesions	Oral squamous cell carcinoma	[63]
	Induction of autophagy	Lymphoma	[36]
	Inhibits growth and decreases resistance to anoikis	Thyroid cancer	[35, 64]
	Inhibits skin tumor promotion	In overweight and obese mice with papilloma and squamous cell carcinoma	[65]
	Suppresses HER2 oncoprotein overexpression	Breast cancer	[101]
Suppression of IGF signaling	Prevents androgen-mediated IGF-1R up-regulation; reduces cell proliferation, invasion, and clonogenic capacity	Prostate cancer cells	[82]
	Reduces the circulating levels of insulin and IGF-1; blocks cell growth and proliferation	A tobacco carcinogen-induced lung cancer model in A/J mice	[60]
AMPK-induced phosphorylation of insulin receptor substrate-1	Switches off IGF-1-induced activation of Akt/Tsc1/mTOR	Human pancreatic cancer cells, breast cancer cells	[83–85]
Activation of AMPK	Disruption of crosstalk between insulin/IGF-1R and GPCR signaling	Pancreatic cancer	[86]
Activation of the JNK/p38 MAPK pathway	Apoptosis-mediated effect	Lung cancer cells	[100]
The MAPK signaling pathway	Synergistic effects of metformin in combination with gefitinib	Lung cancer	[59, 91]
	Blocks tumor cells migration and invasion and inhibits MMP-9 activation	Human fibrosarcoma	[92]
	Inhibits cell growth and colony formation and induces cell cycle arrest	Breast cancer	[93–96]
	Blocks survival signals	Prostate cancer	[97]
		Endometrial cancer	[98]
Inhibition of the NF-κB pathway	Halts proliferation of cancer cells and causes death; sensitizes to chemotherapeutic reagents	Inflammation-associated tumors	[107]
Repression of the NF-κB and mTOR signaling pathways	Growth inhibition	Cutaneous squamous cell carcinoma	[99]
Inhibition of CSCs	Inhibits cellular transformation and selectively kills cancer stem cells	Preclinical breast cancer models	[119]
Down-regulation of CSC markers	Inhibits cell proliferation, migration, and invasion	Pancreatic cancer	[121, 122]
Targeting CSCs and mTOR	Inhibits esophageal cancer cell growth and sensitizes cells to 5-FU cytotoxic effects	Esophageal cancer cells	[123]
Selective suppression of NF-κB nuclear localization and STAT3 activity	Inhibits nuclear translocation of NF-κB and phosphorylation of STAT3 in CSCs	Breast cancer, prostate cancer, and melanoma cell lines	[126]

*AMPK* adenosine monophosphate-activated protein kinase, *REDD1* regulated in development and DNA damage 1, *mTOR* mammalian target of rapamycin, *HER2* human epidermal growth factor receptor-2, *IGF* insulin-like growth factor, *GPCR* G protein-coupled receptor, *IGF*-*1* insulin-like growth factor-1, *JNK* c-Jun N-terminal kinase, *MAPK* mitogen-activated protein kinase, *MMP*-*9* matrix metallopeptidase-9, *NF*-*κB* nuclear factor kappaB, *CSCs* cancer stem cells, *5*-*FU* 5-fluorouracil, *STAT3* signal transducer and activator of transcription 3

### Activation of adenosine monophosphate-activated protein kinase (AMPK)

#### AMPK-dependent effects of metformin

Activation of adenosine monophosphate-activated protein kinase, an intracellular energy sensor, is activated by elevating the ratio of adenosine monophosphate (AMP)/adenosine triphosphate (ATP). Once activated, AMPK restores cellular energy levels by inhibiting anabolic processes and promoting catabolic processes, e.g., glycolysis and fatty acid oxidation, to increase the AMP/ATP ratio [27, 28]. Vazquez-Martin et al. [29] reported that activation of AMPK inhibited cell mitosis and proliferation by directly influencing the dynamics of cell division during mitosis. Metformin has also been reported to exert its antineoplastic effects by stimulating AMPK [30–32] through up-regulation of the p53–p21 axis and down-regulation of cyclin D1 levels. Metformin inhibits the corresponding cyclin-dependent kinases and then induces G_1_-phase arrest of the cell cycle [33]. Moreover, Kisfalvi et al. [34] reported that metformin caused sustained and significant increases in AMPK activity through Thr^172^ phosphorylation and that the specific AMPK inhibitor compound C attenuated the effect of metformin on DNA synthesis, revealing an AMPK-dependent pathway for metformin treatment of pancreatic cancer. However, Klubo-Gwiezdzinska et al. [35] observed different results that AMPKα knockdown by small interfering RNA (siRNA) and compound C did not prevent the growth-inhibitory effects of metformin on medullary thyroid cancer cells. Shi et al. [36] observed that both molecular and pharmacologic knockdown of AMPK counteracted the metformin-induced growth inhibition and G_0_/G_1_ cell cycle arrest of lymphoma cells. Shi et al. [36] reported that AMPKα siRNA caused not only a striking attenuation of the lymphoma cell response to metformin but also a further growth inhibition when it was combined with doxorubicin. Moreover, in acute lymphoblastic leukemia (ALL), knockdown of AMPKα by short hairpin RNA (shRNA) rescued cells from metformin-induced apoptosis, which was associated with restoration of the unfolded protein response (UPR)/glucose-regulated protein 78 kDa (GRP78) function, down-regulation of UPR apoptotic markers inositol-requiring enzyme 1α (IRE1α) and C/EBP homologous protein (CHOP), and interruption of protein synthesis. Studies on breast cancer therapy have demonstrated that inhibition of AMPK with siRNA decreased the suppression of multidrug resistance 1 (*MDR1*) gene activation after exposure to metformin [37]. Furthermore, overexpression of a dominant-negative mutant of AMPK attenuated the inhibitory effects of metformin on the phosphorylation of cAMP-responsive element-binding protein (CREB) and the expression of MDR1 [37]. Taken together, the antidiabetic drug metformin exhibits anticancer effects that are associated with activation of the AMPK signaling pathway.

#### AMPK-independent effects of metformin

In contrast to the above findings, Sahra et al. [38] reported that the anti-proliferation effect of metformin was independent of the AMPK pathway. They used AMPK siRNA to inhibit the two catalytic subunits of AMPK, but AMPK inhibition did not block the G_0_/G_1_ cell cycle arrest induced by metformin. Their subsequent study showed that a negative regulator of mammalian target of rapamycin (mTOR), regulated in development and DNA damage 1 (REDD1), mediated the effects of metformin on the cell cycle arrest and cyclin D1 alteration [39]. Similarly, Yasmeen et al. [40] found that metformin-induced apoptosis of human ovarian cancer cells was independent of AMPK. In addition, AMPK deficiency sensitized cancer cells to the growth-inhibitory effects of metformin [41]. Arai et al. [42] demonstrated that metformin-mediated repression of chronic inflammatory responses was associated with inhibition of tumor necrosis factor alpha (TNFα) production in human monocytes, an event that was most likely independent of AMPK activation. Chronic inflammation may provide a basis for cancer progression, but there was no obvious change in phosphor-AMPKα observed after metformin treatment [43]. Collectively, these studies provide compelling evidence that certain antitumor effects of metformin are independent of the AMPK signaling pathway [38–43].

### Inhibition of the mTOR pathway

mTOR plays a critical role in regulating cellular energy homeostasis by modulating cellular processes such as protein synthesis and autophagy [44–47]. mTOR signaling exerts significant positive regulation of cell proliferation and tumorigenesis in diverse cancers, and it is frequently aberrantly activated in cancers. Activation of mTOR is associated with malignant tumor progression, resistance to chemotherapy and molecularly targeted therapies, and dismal prognosis [48–52]. mTOR is involved in the formation of two functionally and biochemically discrete signaling complexes: rapamycin with either nutrient-sensitive mTOR1 or nutrient-insensitive mTOR2 [53]. Components upstream of mTOR1 include tuberous sclerosis complex 1 (TSC1) and 2 (TSC2) [54, 55]. The combination of TSC1 and TSC2 functions as a tumor inhibitory complex that suppresses mTOR activity. Such mTOR signaling suppression reduces the phosphorylation of major downstream substrates, such as the eukaryotic initiation factor 4E-binding protein 1 (4E-BP1), ribosomal protein S6 kinase (S6K), and initiation factor eIF4G, and net inhibition of global protein synthesis and proliferation in a large number of cancers [56–58].

Metformin-induced inhibition of the mTOR pathway has been demonstrated in different types of cancer, such as leukemia [30, 32, 59, 60], lung cancer [61, 62], breast cancer [63, 64], oral squamous cell carcinoma [65], lymphoma [36], and thyroid cancer [35, 66] in human, as well as in both papilloma and squamous cell carcinoma in mice [67]. Metformin induces the liver kinase B1 (LKB1)-mediated activation of AMPK, which in turn blocks mTOR signaling and protein synthesis in many cancer cell lines [58, 68, 69]. AMPK impacts mTOR through phosphorylation and activation of the tumor suppressor TSC2, which results in inhibition of a downstream small GTPase (RHEB), negatively regulating mTOR activity [70, 71]. In contrast, metformin can also inhibit mTOR in an AMPK-independent pathway by reducing the levels of insulin-like growth factor-1 (IGF-1) [72, 73]. Kalender et al. [74] have shown that the inhibitory effects of metformin on mTOR signaling were mediated by Rag GTPases in the absence of AMPK and TSC1/2. Of note, one study indicated that metformin directly influenced mTOR in a p53-dependent manner through an AMPK-independent mechanism to boost the level of REDD1, a negative regulator of mTOR [39]. In that report, REDD1 inactivation, using siRNA or REDD1^−/−^ cells, abrogated cell cycle arrest independently of AMPK.

### Suppression of the IGF signaling pathway

Insulin and IGFs are key regulators of metabolism and growth. A rapidly growing body of researches has revealed that insulin and IGFs are associated with cancer progression by activating signaling pathways that are associated with cell growth and proliferation [75]. There are two subtypes of IGF, IGF-1 and IGF-2, which are both mitogenic and antiapoptotic. IGF-1 receptor (IGF-1R) binds to the ligand IGF-1, IGF-2, or insulin to promote autophosphorylation of tyrosine at its kinase domain. This triggers tyrosine and serine phosphorylation to form binding sites for insulin receptor substrates (IRSs) and Src and concomitant activation of signaling through the phosphatidylinositol-3-kinase (PI3K)/Akt/mTOR and RAS/RAF/mitogen-activated protein kinase (MAPK) pathways [76–78]. Moreover, overexpression of IGF-1R can induce tumor formation and metastasis [79, 80]. Likewise, in endometrial cancer cells, overexpression of IGF-1R triggers endometrial hyperplasia and contributes to type I epithelial cell growth by activating PI3K/Akt/mTOR signaling [3, 81, 82]. In addition, activation of IGF accelerated YYH1 tumor progression by promoting vascular smooth muscle cell proliferation, migration, and angiogenesis [83].

Emerging evidence suggests that metformin can exert its anticancer functions by reducing the levels of IGF-1. Metformin, which acts as an insulin-sensitizing agent, decreases IGF-1 by indirectly down-regulating insulin and insulin-binding proteins to reverse hyperinsulinemia, which may be a mechanism for metformin’s anticancer effects [75]. In fact, Memmott et al. [62] observed that metformin acted by reducing the circulating levels of insulin and IGF-1 to block tumor growth and proliferation in a tobacco carcinogen-induced lung cancer model in A/J mice. Similarly, Malaguarnera et al. [84] recently confirmed that metformin reduced cell proliferation, invasion, and clonogenic capacity by preventing the androgen-mediated up-regulation of IGF-1R. Moreover, recent studies have shown that metformin-mediated activation of AMPK increased the phosphorylation of IRS-1, diminishing the IGF-1-induced activation of Akt/TSC1/mTOR [85–87]. Another mechanism relevant to IGF-1 could be disruption of the crosstalk between insulin receptor/IGF-1R and G protein-coupled receptor (GPCR) signaling via metformin-induced activation of AMPK [86, 88].

### Inhibition of other signaling pathways

#### Metformin and the JNK/p38 MAPK pathway

Other possible mechanisms for the beneficial effects of metformin on cancer development have also been described. The MAPK-involved pathways are significant intracellular signaling pathways that regulate cell growth, differentiation, proliferation, apoptosis, and migration [89–92]. Four major MAPK pathways have been described: extracellular signal-regulated kinase (ERK, also known as p42/44 MAPK), big MAP kinase (BMK, also known as ERK5), p38 MAPK (also known as SAPK2/RK), and c-jun N-terminal kinase (JNK, also known as stress-activated protein kinase 1 [SAPK1]) pathways. Although not universally observed in all cells, metformin has been found to be relevant to MAPK signaling in certain malignancies such as lung cancer [61, 93], human fibrosarcoma [94], breast cancer [95–98], prostate cancer [99], endometrial cancer [100], and cutaneous squamous cell carcinoma [101]. Metformin has been shown to exert an apoptosis-mediated effect through activating the JNK/p38 MAPK pathway and enhancing expression of growth inhibition and DNA damage-inducible gene 153 (GADD153) [102]. Monteagudo et al. [99] used a dendrimer-vehiculized siRNA to block the MAPK signaling pathway and found that the blockade enhanced the anticancer effect of metformin. Other data from Tseng et al. [93] suggested that metformin could reduce paclitaxel-induced, p38 MAPK-mediated expression of excision repair cross complementary 1.

#### Metformin and the HER2 pathway

Human epidermal growth factor receptor-2 (HER2) belongs to the epidermal growth factor receptor family, the members of which possess tyrosine kinase activity. HER2 is overexpressed in approximately 20%–30% of breast cancers. As a significant biomarker of breast cancer, HER2 is a crucial therapeutic target in breast cancers that overexpress HER2. Vazquez-Martin et al. [103] studied the effects of metformin on cultured human breast cancer cells with HER2 amplification and observed that ectopic overexpression of the *HER2* oncogene significantly enhanced metformin-induced growth inhibition. They also reported that metformin suppressed HER2 oncoprotein overexpression via AMPK-independent inhibition of mTOR in human breast cancer cells [103]. Interestingly, metformin notably blocked HER2 tyrosine kinase activity at low therapeutic concentrations [96]. In addition, it was found that metformin combination therapy with the anti-HER2 monoclonal antibody trastuzumab could eliminate stem/progenitor cell populations in *HER2*-amplified breast carcinoma cells [104].

#### Metformin and the NF-κB pathway

Nuclear factor kappaB (NF-κB) is a protein complex that functions as a signal-induced transcription factor to regulate proliferation and apoptosis [105]. It is an important potential target in cancer therapy [106–108]. Inhibition of NF-κB can induce cancer cells to halt proliferation and die or can sensitize cells to chemotherapeutic reagents [109]. Kim et al. [37] reported that metformin activated AMPK and inhibited mTOR by suppressing NF-κB and CREB. Later, Chaudhary et al. [101] observed that the growth-inhibitory effect of metformin repressed the NF-κB and mTOR signaling pathways. Additionally, Zheng et al. [110] showed that metformin dampened NF-κB signaling by boosting NF-κB inhibitor alpha (IκBa) in hepatocellular carcinoma cell lines. Moreover, forced expression of p65 or overexpression of an undegradable mutant form of IκBa was found to activate NF-κB signaling, thereby attenuating the antitumor effects of metformin.

### Metformin targets cancer stem cells

Cancer stem cells, also called tumor-initiating cells, are a subset of cancer cells that are believed to have indefinite potential capacity to self-renew and result in tumorigenesis [111]. Compared with non-cancer stem cells, CSCs are both chemoresistant [112–116] and radioresistant [116–119]. CSCs are compelling candidates for tumor origination and may contribute to cancer metastasis and relapse, which are the main impediments to prolonging overall survival. Of note, self-renewal and inherent chemoresistance are responsible for tumor recurrence [120]. Therefore, development of non-toxic treatment strategies targeting CSCs will be of significant therapeutic benefit.

Metformin inhibition of CSCs was first demonstrated in 2009 in preclinical breast cancer models [121]. Subsequent reports indicated that metformin improved the response of human cancer xenografts to conventional chemotherapy by eradicating CSCs in multiple cancer types [104, 122]. In parallel, metformin down-regulates CSC marker genes in pancreatic cancer [123, 124], esophageal cancer [125], and breast cancer [126]. In pancreatic cancer, metformin inhibits cell proliferation, migration, and invasion by weakening CSC function mediated by deregulating miRNAs [123]. In esophageal cancer, metformin inhibits esophageal cancer cell growth and sensitizes cells to the cytotoxic effects of 5-fluorouracil (5-FU) by targeting CSCs and mTOR [125]. Regarding the mechanisms by which metformin targets CSCs, Song et al. [127] reported that metformin increased the sensitivity of cancer cells to radiotherapy and exhibited cytotoxicity toward CSCs, overcoming their radioresistance via activation of AMPK and suppression of mTOR. In contrast, Hirsch et al. [128] reported that metformin selectively suppressed NF-κB nuclear localization and Stat3 activity in CSCs.

## Conclusions

In conclusion, in vitro and in vivo studies strongly indicate that metformin, a widely prescribed oral medication used as front-line therapy for type 2 diabetes, could be a valuable adjuvant therapy for cancer. Metformin may become a useful adjuvant drug in association with established anticancer therapies, and there are multiple clinical trials examining the effects of metformin on cancer outcomes. In general, most data support the hypothesis that metformin is protective against cancer. However, based on the current preliminary findings, it appears that metformin is not an effective treatment alone for unselected patient populations or larger number of patients. Therefore, we recommend that combination therapies with metformin as well as potential novel biomarkers that could identify patient populations sensitive to metformin treatment should be pursued. Further studies are needed to improve our understanding of the pathways linking high metformin efficacy and cancer development.

Overall, the biological effect of metformin on cancer cells is based on its ability to activate AMPK or inhibit downstream growth factor signaling through inhibition of mTOR. Metformin also has indirect effects on the IGF and JNK/p38 MAPK pathways; other possible mechanisms include inhibition of the HER2 and NF-κB signaling pathways. Further support for these observations is that metformin kills cancer stem cells and changes the properties of CSCs. Nonetheless, a large number of further translational studies are required to evaluate the potential of metformin as an additive antitumoral agent.
